# The clinical features, management options and complications of paediatric femoral fractures

**DOI:** 10.1007/s00590-021-02933-1

**Published:** 2021-04-11

**Authors:** Sean Duffy, Yael Gelfer, Alex Trompeter, Anna Clarke, Fergal Monsell

**Affiliations:** 1 Severn Deanery, Bristol, UK; 2 St George’s Hospital, London, UK; 3 Bristol Children’s Hospital, Bristol, UK

**Keywords:** Paediatric, Femur, Fracture, Review, Treatment, Complications

## Abstract

This article discusses the incidence, applied anatomy and classification of paediatric femoral fractures based on critical appraisal of the available evidence. The aim is to identify techniques that are relevant to contemporary practice whilst excluding the technical details of individual procedures that are beyond the scope of this review. Injuries of the proximal, diaphyseal and distal segments are considered individually as there are considerations that are specific to each anatomical site. Femoral neck fractures are rare injuries and require prompt anatomical reduction and stable fixation to minimise the potentially devastating consequences of avascular necrosis. Diaphyseal fractures are relatively common, and there is a spectrum of management options that depend on patient age and size. Distal femoral fractures often involve the physis, which contributes up to 70% of femoral length. Growth arrest is common consequence of fractures in this region, resulting in angular and length-related deformity. Long-term surveillance is recommended to identify deformity in evolution and provide an opportunity for early intervention. Deliberate injury should be considered in all fractures, particularly distal femoral physeal injuries and fractures in the non-walking child.

## Introduction

This article critically appraises the published evidence related to the paediatric patient with a femoral fracture, evaluating the proximal, diaphyseal and distal segments separately. The incidence, applied anatomy, classification and contemporary management strategies are discussed. Proximal and distal femoral fractures are less common, but management tends to be technically difficult, with considerable complication profiles, whilst diaphyseal fractures are more common and the treatment is dependent on the age and size of the child.

## Proximal femur

Femoral neck fractures are rare injuries in children and account for approximately 1% of all paediatric fractures [1, 2].

These are associated with a high complication rate, including avascular necrosis (AVN) and mal-union, often with devastating long-term consequences [3]. This group of fractures often demands an aggressive management strategy [2] and treatment should be aimed at achieving rapid, anatomic reduction with stable internal fixation [3, 4].

### Epidemiology

Most paediatric femoral neck fractures are caused by high-energy trauma, typically involving motor vehicle accidents and falls [5, 6].

The rate increases with age and neck fractures account for 7.0% of all femoral fractures in patients under two years and 12.8% in patients aged between 13 and 18 years [7].

Fractures may occur following low energy or seemingly trivial injury, particularly in association with local pathology including bone cysts and fibrous dysplasia [8]. Proximal femoral fractures have also been recognised as an atypical presentation of deliberate injury, particularly in children who are not yet walking [9, 10].

### Regional anatomy

#### Ossification

The primary ossification centre of the proximal femur appears between the fourth and seventh months of life. The secondary centres representing the greater trochanter appear between two and four years and the lesser trochanter at the time of puberty. The proximal femoral physeal plate contributes to approximately 30% of the overall length of the femur and 13% to the entire limb. All centres fuse between the ages of 14 and 18 years [11].

Interruption of the vascular supply of the femoral head may result in AVN [12–14] and vascular interruption of the physis, in the younger child, may cause growth arrest and result in progressive proximal femoral deformity [15].

#### Vascular anatomy

The development of the vascular supply of the proximal femur follows a predictable sequence, and an appreciation of the details is required for a rational treatment strategy for hip fractures in the developing skeleton [16, 17]. From birth to formation of the primary ossification centre at four–six months, the cartilaginous epiphysis of the proximal femur is supplied by the medial femoral circumflex artery (MFCA), lateral femoral circumflex artery (LFCA) and to a lesser degree, the artery of ligamentum teres. After ossification of the femoral head, branches from the LFCA are prevented from crossing the growth plate and the inferior and superior retinacular branches of the MFCA supply the epiphysis [17, 18]. After skeletal maturity, branches of the MFCA and LFCA form an extracapsular anastomosis in the intertrochanteric region, with branches supplying the metaphysis and epiphysis. The MFCA remains the dominant supply to the femoral head with a less important contribution from LFCA and the artery of ligamentum teres [18].

### Classification

Delbet described a system for classifying adult femoral neck fractures in 1928 [19]. This was modified by Collona in 1929 for use in children [20], dividing fractures into four subtypes: type I involving the physis (AVN 38%), type II transcervical (AVN 28%), type III basicervical (AVN 18%) and type IV intertrochanteric (AVN 5%) [21]. This is a useful predictor for AVN [21–23] and is widely used in contemporary paediatric practice. A meta-analysis conducted by Moon and Mehlman reported an increasing rate of AVN associated with more proximal subtypes [21].

The Müller-AO system modified in 2006 accounts for paediatric-specific patterns and is commonly used [24]. It provides a comprehensive system for accurately characterising proximal femoral fractures, including epiphyseal and metaphyseal patterns.

### Imaging

Plain anteroposterior (AP) and lateral radiographs of the affected femur are usually sufficient for initial diagnosis [14], but there should be a low threshold for obtaining either CT or MRI to define the pattern of femoral neck fractures and this is fundamental to planning the surgical approach and stabilisation in fractures with intra-articular extension [25].

### Surgical strategy

Whilst there is general agreement that surgical stabilisation is associated with lower rates of AVN [23], there is lack of consensus concerning the optimum treatment for this group of injuries. This is in part due to the low incidence of these injuries and therefore the paucity of individual experience.

Surgical management should generally avoid further injury to the growth plate with anatomical reduction of fragments and stabilisation with pins or screws allowing early protected weight-bearing, therefore minimising potential complications particularly AVN [26].

Fractures close to, or involving, the growth plate (Delbet type I/II) should, however, be treated with primacy given to stability over iatrogenic injury to the physis [3].

Yeranosian et al. reported a systematic review 30 studies, comprising 935 patients and reported that fractures managed with closed indirect reduction under radiological control were associated with lower AVN rates. This may, however, have been influenced by the predominance of open reduction in type I fracture [23].

Ju et al. [27] reported a lower incidence of AVN and better outcomes with open reduction compared to closed reduction and internal fixation in a series of 58 children with displaced femoral neck fractures.

**Fig. 1:**
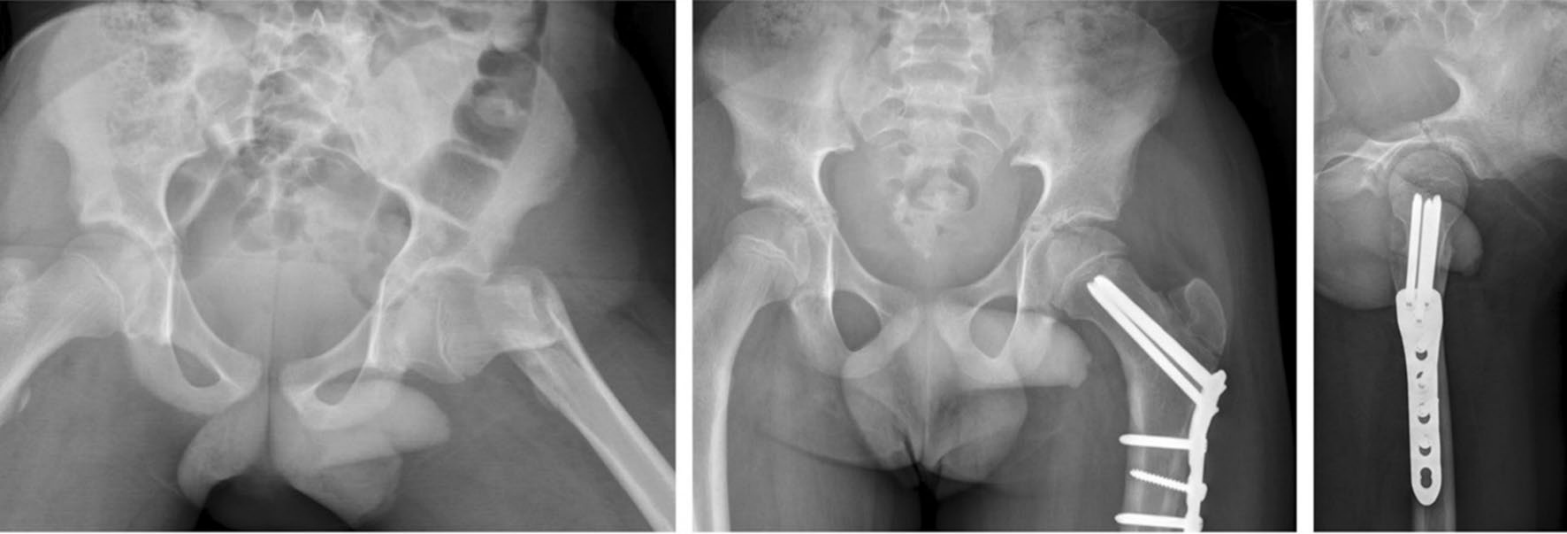
Delbet type III fracture anatomically reduced and stabilised with a fixed angle locking plate

In an observational study of 239 fractures, Wang et al. highlighted the need for stable fixation, reporting significantly reduced AVN rates with femoral locking plates compared to other forms of fixation including cannulated screws or Kirshner wires in a population predominated by type II and III fractures (67.6% and 29.9%, respectively) [14]. Paediatric and adolescent dynamic hip screw constructs have been used, particularly in older children, but newer generation locking plates are now available and provide a fixed angle construct with superior fracture stability (Fig. 1).

A complete description of all available surgical options is beyond the scope of an article of this type and detailed information on fixation options is provided in the AO surgical reference (Paediatrics) [28].

Intracapsular haematoma is implicated in the development of AVN due to the effect of external compression on the retinacular circulation, with some authors recommending surgical decompression in all cases [29–31]. This procedure is technically straightforward and has a low complication rate, but the beneficial effects have not unanimously been reported [23].

There is a longstanding and continuing debate on the optimum timing of reduction and stabilisation. It is axiomatic that prompt reduction of a displaced proximal femoral fracture will reduce the risk of femoral head ischaemia by re-establishing circulation, but there is lack of consensus. Stone et al. reported reduced AVN rates with early reduction [32], replicating the findings of earlier studies [4, 6, 23, 33].

Yeranosian et al. reported a systematic review that identified a 4.2 increase in AVN rate when definitive treatment was delayed > 24 h [23]. Wang et al. demonstrated that age and initial displacement were independent risk factors for AVN and did not identify an association between the time from injury to treatment [14]. Alkhatib et al. conducted a systematic review that considered six cohort studies involving 231 patients and did not identify a statistically significant difference between early (< 24 h) and late (> 24 h) treatment [22].

### Authors’ approach

The authors’ preferred management of displaced fractures is with open reduction using a Watson-Jones approach, anatomical reduction and stabilisation with a fixed angle locking plate. While we recognise that the unambiguous case for early intervention has not been made, we recommend surgical decompression and stabilisation within 12 h of injury.

### Complications

#### Avascular necrosis

Avascular necrosis (AVN) represents the primary determinant for long-term outcome after paediatric hip fracture, usually presenting within one year of injury [12–14]. Higher incidence is associated with more proximal fracture patterns [21] and the degree of initial displacement is also an important consideration [14]. Alkhatib et al. identified a significant relationship between AVN rates and displacement/Delbet fracture type, with displaced type I and II fractures associated with the highest risk (OR 3.8 and 2.4, respectively) [22]. Moon et al. reported a 1.14 increased risk per year of age [21] and Wang et al. identified age over 12 years as a significant independent risk factor [14].

#### Non-union

The rate of non-union is reported between 6 and 33% of all paediatric hip fractures, with higher rates in older papers, perhaps reflecting progress in the techniques of fracture fixation [34–37].

Non-union is generally seen after mid and basal cervical fractures and is often due to failure to obtain or maintain an anatomic reduction, in addition to an unfavourable fracture configuration. CT imaging may assist with identification, which should be managed with subtrochanteric valgus osteotomy, with bone grafting reserved for recalcitrant cases [3].

#### Mal union

Coxa vara has a reported incidence between 20 and 30% and while commonly asymptomatic, may also require realignment osteotomy, particularly in older children [2, 36, 38, 39].

#### Growth arrest

Premature physeal closure has been reported in 5 to 65% [2, 36, 38, 39] but as the proximal femoral physis contributes 13% of overall longitudinal growth of the limb, shortening due to premature growth arrest is not usually a clinical issue, except in very young children. This should form part of the post-injury surveillance and may require surgical equalisation, usually with contralateral distal femoral epiphysiodesis.

## Femoral shaft

### Epidemiology

Diaphyseal femoral fractures in children have a bimodal distribution with peaks of incidence in patients aged two and 17 years and are greater than 2.5 times more frequent in boys [40, 41].

The largest UK study reported >3000 femoral fractures in children aged < 16 years and observed that the incidence decreased from 0.33 to 0.22/1000/year between 1991 and 2001 [42].

Loder et al. [7] reported a database review of approximately 10,000 femoral fractures and provided an overview of the patient characteristics in a developed industrial environment. Motor vehicle collision was implicated in 35% and accounted for the largest percentage in older children, particularly adolescents. Falls were responsible for 33% and were most common in children aged less than 6 years [7].

Non-walking is the single best predictor for non-accidental injury (NAI) [43], but the presence of a femoral fracture in a child requires assessment for deliberate injury, irrespective of age and ambulatory status. An epidemiological study of 1358 fractures performed in the UK reported a deliberate injury rate of 3.8%, of which 91% occurred in children under two years [40].

### Surgical strategy

Management decisions are primarily based on the age and size of the patient. Other important considerations include fracture configuration, surgeon experience and disruption to family life.

**0–6 months**

Femoral fractures in this age group heal rapidly, and a short period of non-invasive immobilisation is sufficient for the majority, with gallows traction suitable for patients < 10–15 kg [44].

This can be used as definitive management or with elective substitution for a hip spica, either immediately or as a delayed event [45]. Pavlick harness is also commonly used in this age group, particularly in the neonate with a birth fracture [46].

**6 months–5 years**

Non-invasive treatment is also the recommended treatment for femoral fractures in this age group. Immediate traction provides fracture stability and analgesia that facilitates comfortable transport [47–52]. The type of traction is age and size dependant, with inline skin traction, Thomas’ splint and balanced traction in common use [52–54].

Conversion to a hip spica is typically performed on the next available operating list but delayed casting may reduce the incidence and extent of mal-union [44]. Mal-union is rarely an issue in this age group due to the remodelling potential in the young child. Shortening is inevitable in most fracture patterns, but overgrowth is common and clinically relevant limb length discrepancy is unusual [45].

Whilst non-invasive treatment is conventional, Gordon et al. reported a prospective multicentre study, which demonstrated a reduced impact on family life with elastic stable intramedullary nails (ESIN) compared to hip spica for children under six, with no statistically significant difference in analgesia requirement or healing outcomes [55].

**5–16 years**

Non-operative management is possible in this age group, but this requires prolonged inpatient, or domiciliary traction and due to the associated social and financial imperatives, operative management has become the treatment of choice in the industrialised world.

**Fig. 2:**
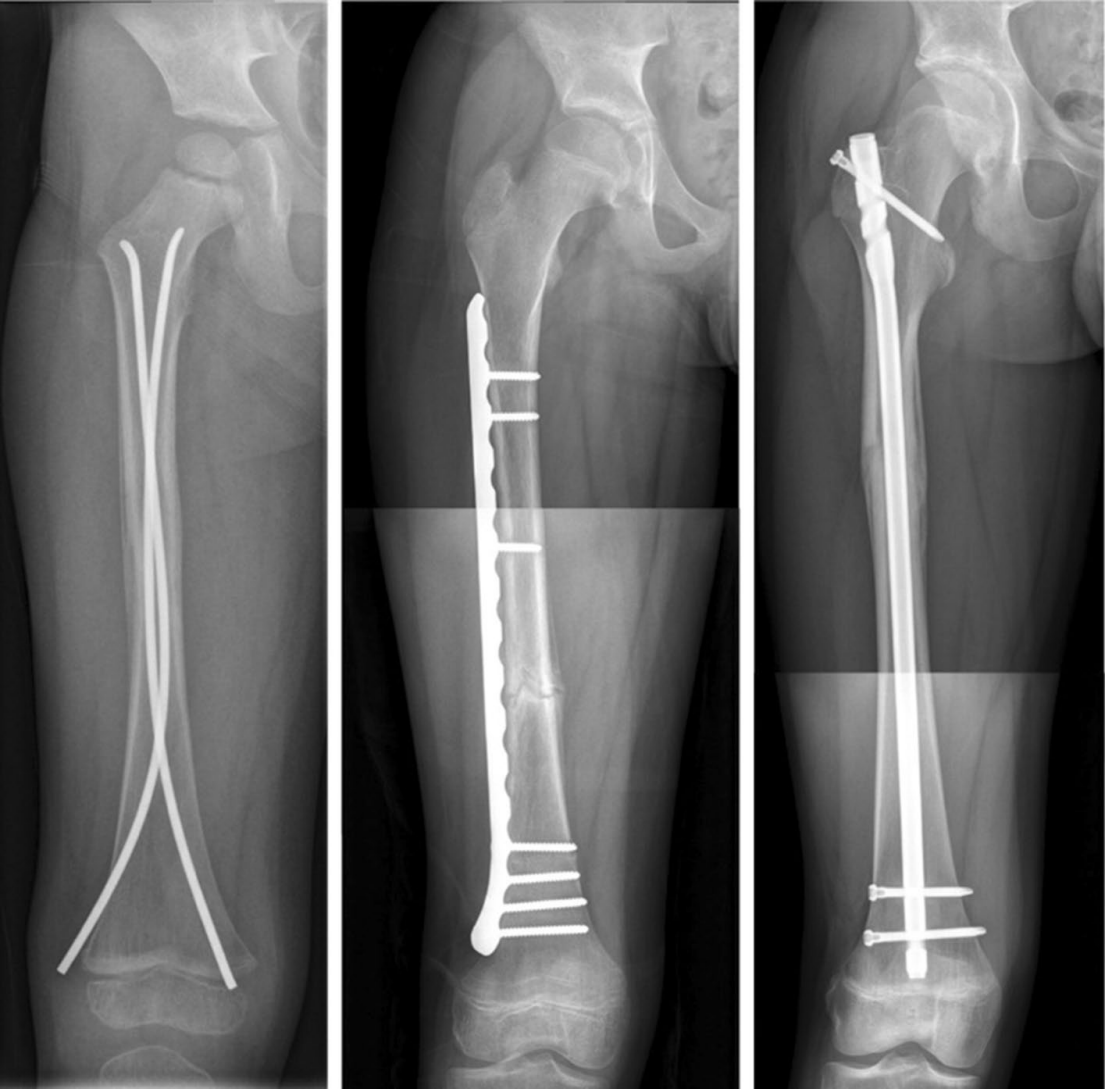
Femoral shaft fractures treated with ESIN (left), MIPO (middle) and a rigid intramedullary nail (right)

There is no consensus regarding the optimal surgical management and techniques relevant to this age group include the use of ESIN, rigid intermedullary nails, open or minimally invasive plates (Fig. 2) and external fixators.

#### Elastic stable intramedullary nails

There are a number of studies reporting good or excellent outcomes for paediatric femoral fractures treated with ESIN using stainless steel or titanium implants [56–59].

Titanium is more flexible, and the elasticity is fundamentally important for maintenance of reduction and enhancement of fracture healing. A simple transverse mid-diaphyseal fracture is a strong indication, and fractures of the proximal and middle third are commonly approached with a retrograde technique. Antegrade nailing is technically more straightforward in the distal third, as distal entry points are close to the fracture site and the nail configuration is not sufficiently stable [28]. Flexible nailing can be used for long oblique and spiral fractures but Narayanan et al. and Sink et al. reported an increased risk of shortening and mal-union in length unstable fractures [60, 61] and the addition of end caps improves axial stability in suitable fractures [62, 63].

Anatomical reduction is not necessary and Wallace et al. demonstrated that remodelling of up to 25 degrees sagittal and coronal angulation was possible [50]. Femoral malrotation is common following ESIN fixation with a reported incidence up to 41.6% [64]. Careful intraoperative assessment is required to avoid rotational asymmetry, which has poor remodelling potential [51].

This technique is commonly used in Europe and North America and provides excellent results with a low complication profile in patients under 50 kg, independent of the fracture pattern [65]. Some authors report age as the principle factor that determines the choice of treatment and recommends operative management for fractures in patients aged four and older [53]. Others consider that weight is more important and use ESIN in children < 49 kg and rigid nailing or plating in heavier children, with the choice of implant dependant on the fracture pattern [44, 66].

#### Rigid intramedullary nails

Piriformis entry nailing systems are associated with AVN in the developing skeleton with a reported incidence of 1–5% [67–69] and are generally avoided in this age group. Lateral entry nails are designed to avoid injury to the femoral head blood supply with no cases of AVN reported in two series of 246 patients and 78 patients and one systematic review of 19 papers [67, 70, 71].

Moroz et al. compared locked rigid nail systems to ESIN and reported a reduced rate of mal-union with rigid nails in children weighing > 49 kg, irrespective of fracture configuration [66]. Garner et al. also categorised patients according to weight and fracture type and did not detect a significant difference in mal-union between rigid nails and ESIN with length stable fractures, in patients with a mean body weight of 60 kg [72].

#### Plating

Open or submuscular plating is an option for high energy, multifragmentary injuries in skeletally immature patients with fractures that are unsuitable for flexible nailing, due to anatomical location, fracture pattern or patient weight. The development of minimally invasive plating systems has popularised this technique in the management of paediatric femoral fractures. A retrospective review of 344 children treated with submuscular plating, rigid nailing or flexible nailing reported an earlier return to full weight bearing and union in the plating group [73]. Spiral fractures were more frequent in the plating group and this may have contributed to the faster time to union in this study. There is a paucity of high-level studies on submuscular plating in the paediatric population; however, favourable outcomes have been reported in retrospective series [74, 75].

#### External fixation

External fixation a useful technique in patients with; open fractures, high energy multifragmentary injuries, polytrauma or injuries requiring transfer to another centre. Ease of application is an advantage and provides effective reduction and stabilisation in the short term, with minimal additional blood loss and avoidance of the zone of injury. Bar-On et al. conducted a randomised trial comparing flexible nails with external fixators for definitive fixation and reported significantly improved clinical and radiographic outcomes in the flexible nail group. The authors recommended that external fixators should be reserved for open and multifragmentary injuries [76].

### Skeletally mature patients

Displaced femoral shaft fractures in adolescents with closed proximal femoral growth plates should be treated with an identical approach to the adult patient, with rigid, locked intramedullary nails [69, 77]. Contemporary nailing systems stabilise the femur proximally and distally, controlling rotation and alignment. This permits early rehabilitation in multifragmentary and length unstable fracture patterns.

### Authors’ approach

The authors recommend immediate hip spica for children under four years. We recommend that older children weighing < 50 kg are treated with ESIN but consider MIPO in axially unstable fracture patterns. Older children weighing > 50 kg with an open proximal physis should be treated with lateral entry locked intramedullary nails.

## Distal femur

### Epidemiology

Fractures of the distal femur are rare injuries with a peak incidence between 10 and 12 years of age and are six times more common in males [78, 79]. They are frequently due to sports activities and high-energy mechanisms, particularly motor vehicle accidents and falls [78] with an association between a high-energy mechanism and physeal bar formation [80].

The injury often involves valgus or varus forces to the knee, tensioning the collateral ligaments at the attachment to the distal femoral epiphysis with initial failure of bone, resulting in physeal injuries [81]. The distal femoral physis contributes 70% of femoral length and 40% of overall limb length at an approximate rate of 10 mm per year [82–84].

Distal femoral metaphyseal fractures are associated with a high rate of deliberate injury in non-walking children, with a reported rate of 50% [85] and corner fractures at this location are generally accepted as an indicator of abuse in a child of this age [86–88].

### Classification

The Salte–Harris (SH) classification is the most widely used system [82] and is a significant predictor for outcome [79]. Eid et al. [78] reported a single centre series of 151 distal femoral physeal injuries with Salter–Harris type I in 26%, type II in 43%, type III in 12.5%, type IV in 14.5% and type V in 4%. Other authors have also identified SH II as the most common pattern, with an incidence of 83% in some series [78–80, 89]. Fractures with this pattern are usually displaced, with a reported incidence between 59 and 84% [79, 89], and are associated with a rate of growth arrest four times that of non-displaced injuries [79, 90, 91].

### Imaging

Plain radiographs are unreliable in defining the degree of displacement in SH III injuries, with MRI or CT, often resulting in a change in management [92]. SH V injuries are also commonly overlooked on initial plain radiographs [78].

### Surgical strategy

There is no consensus about the optimum treatment for displaced fractures involving the physis. A spectrum of management options is available and includes long leg casting, closed manipulation and pinning, cannulated screws, submuscular plating and external fixation.

#### Treatment of non or minimally displaced extra-articular injuries (SH I-II)

Non-operative management with immobilisation in a long leg cast is possible for these injuries, provided they are undisplaced, or can be anatomically reduced [79, 89]. Growth disturbance between 16 and 23% is reported, in spite of the absence of manipulation or surgical fixation [79, 89].

#### Treatment of displaced injuries

The majority of displaced extra-articular (SH I–II) and all intra-articular (SH III–IV) fractures require reduction. This may be possible with closed manipulation, but open reduction is often necessary, with a reported rate of 46% [89]. Cast stabilisation alone is unreliable, with a high rate of loss of reduction in the first 2 weeks and re-manipulation associated with a lower success rate [78].

Displaced SH I injuries require internal stabilisation with a physeal-crossing technique [93].Arkader et al. [79] reported a higher rate of complications when fixation crossed the physis but this difference did not reach statistical significance.

**Fig. 3:**
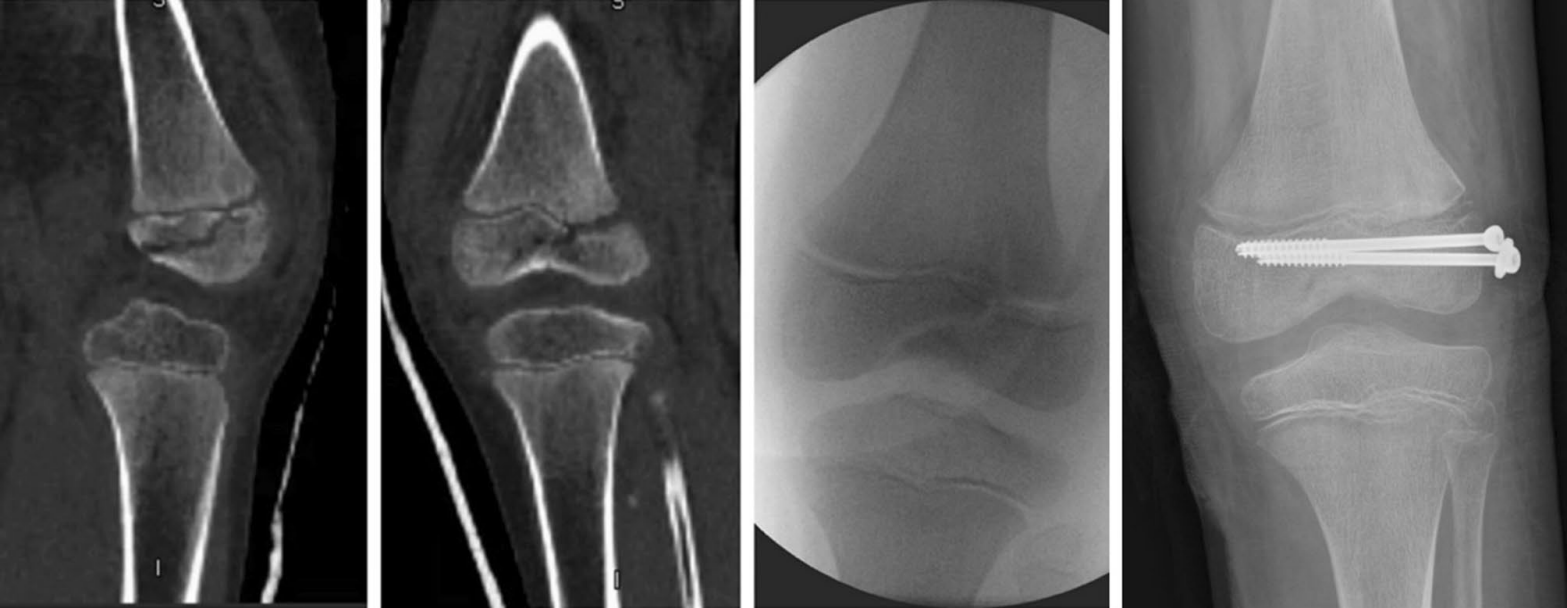
CT scan and intraoperative image demonstrating a SH III fracture with subsequent screw fixation

Garrett et al. reported 55 patients with a median age of ten years with displaced distal femoral physeal fractures, the majority (73%) treated with percutaneous pinning after reduction (Fig. 3).

A physeal bar occurred in 31% in patients with high-energy injuries compared with 5% in those with low-energy injuries. There was a significant association between physeal arrest and increasing severity using the Salter–Harris classification [80].

### Authors’ approach

The authors recommend open reduction and screw fixation for intra-articular fractures. Displaced extra-articular fractures require reduction and stabilisation with metaphyseal screws or crossed smooth wires, determined by the fracture configuration.

### Complications

Distal femoral physeal injuries have an overall complication rate of 40–50% [79, 89, 90]. Rates of 62–90% have been reported in earlier, smaller studies [94, 95], possibly reflecting a less aggressive approach to management. Arkader et al. reported a lower incidence of complications with conservative treatment, but acknowledged selection bias, with surgeons opting for surgical management in more severe injuries [79].

Adams et al. reported an interval study with a modification of their treatment algorithm following a 40% incidence of complications with a conservative approach [89]. They adopted a lower threshold for surgical management in 70 children with an average age of 13. The authors did not demonstrate a significant difference in complications between the conservative and more aggressive surgical groups. In addition, there was no statistical association between all surgical fixation methods and the complication profile. They observed an increased rate in complications with crossed pins compared to metaphyseal screws, but this did not reach statistical significance (*p* 0.067) [89].

#### Growth disturbance

Physeal arrest is the most frequent complication following this injury [79, 89] and when this is associated with an evolving deformity and requires surgical intervention in up to 60% of cases [89].

Basener et al. reported a meta-analysis of 564 fractures that assessed the incidence of growth disturbance according to Salter–Harris subtype. SH 4 fractures were associated with the greatest risk at 64%, with SH 2 58%, SH 3 49% and SH 1 36%. SH 5 fractures were omitted due to insufficient patient numbers for subgroup analysis [90]. Physeal arrest resulted in varus malalignment in 13.9%, valgus in 9.3%, flexion in 12.6% and recurvatum 1.3% [78]. There was limb length discrepancy (LLD) > 1.5 cm in 22% [90] and a higher rate of clinically significant growth arrest following conservative (37%) compared to operative management (27%) [90].

Attempted excision of a physeal bar is often unsuccessful [80] and Arkader et al. [79] reported 55% of patients required contralateral epiphysiodesis or limb lengthening for LLD and epiphysiodesis with osteotomy or osteotomy alone to manage angular deformity.

Implants including a paediatric physeal slide-traction plate have been designed specifically for distal femoral fractures. This minimises the tethering effect of the implant on the growth plate, with favourable results reported in a preliminary study [96].

#### Neurovascular injuries

Peroneal neuropraxia has a reported incidence of 1–7% [78, 79] and invariably recovers spontaneously [78]. Vascular injury caused by popliteal artery compression or injury from displaced distal femoral fractures is rare with a reported incidence of 0–2.6% [78, 79, 89]. The direction of displacement has been reported as significant in some studies [97–99], but without a causal relationship in more recent reports [79, 89].

#### Closing remarks

Paediatric femoral neck fractures are usually associated with a high-energy mechanism. The Delbet classification is a significant predictor for developing AVN, with higher rates associated with increasing fracture proximity to the femoral epiphysis. There is emerging evidence that displacement at presentation, anatomical reduction and fixed angle implant stabilisation reduce AVN rates. Urgent surgery is recommended; however, some studies have failed to identify a statistically significant relationship between the timing of surgical intervention and rate of AVN.

Fractures of the femoral shaft represent the most common femoral fracture and have the most favourable complication profile. NAI should be considered, particularly in the non-walking patient. The management is primarily determined by the age and size of the patient with hip spica, traction or a combination being effective in infants and younger children, while ESIN, plating and rigid intramedullary nailing are more suitable for older and heavier children. Implant choice is dependent on patient weight, fracture configuration, skeletal maturity and surgeon preference.

The distal femoral growth plate contributes the majority of longitudinal femoral growth, and fractures in this region result in growth arrest in up to 50%. A strong association with NAI demands careful investigation, particularly in the presence of a “corner fracture” which is commonly associated with deliberate injury. Surgical management is often required, particularly for displaced fractures and internal fixation provides superior joint reconstruction and stability. There is no good quality evidence to identify the optimum management of these injuries and most series involve small numbers.
