# Correction to: The clinical features, management options and complications of paediatric femoral fractures

**DOI:** 10.1007/s00590-021-02998-y

**Published:** 2021-05-31

**Authors:** Sean Duffy, Yael Gelfer, Alex Trompeter, Anna Clarke, Fergal Monsell

**Affiliations:** 1 Severn Deanery, Bristol, UK; 2 St George’s Hospital, London, UK; 3 Bristol Children’s Hospital, Bristol, UK

## Correction to: European Journal of Orthopaedic Surgery & Traumatology 10.1007/s00590-021-02933-1

The original version of this article unfortunately contained a mistake. Figure 3 was incorrect.

The corrected Fig. 3 is given in the following page.

**Fig. 3:**
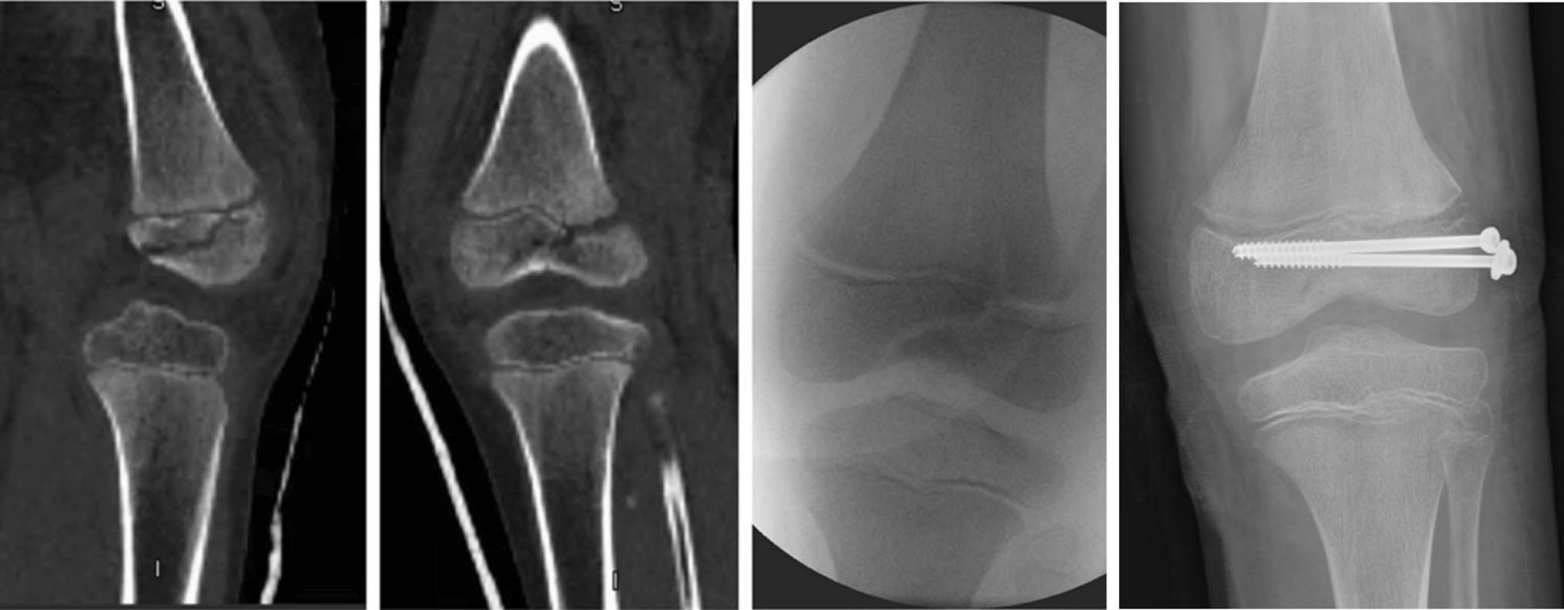
CT scan and intraoperative image demonstrating a SH III fracture with subsequent screw fixation

The original article has been corrected.

